# Development of police law enforcement psychological ability scale and test of reliability and validity

**DOI:** 10.3389/fpsyg.2026.1811849

**Published:** 2026-05-07

**Authors:** Yuliang Feng, Chao Chen, Sen Li

**Affiliations:** 1 Police Training Department, Shanghai Police College, Shanghai, China; 2 Physical Education College, Dalian University, Dalian, Liaoning, China; 3 School of Physical Education and Health, Shanghai Lixin University of Accounting and Finance, Shanghai, China

**Keywords:** law enforcement, psychological ability, evaluation, cognition, emotion, volition

## Abstract

**Introduction:**

The psychological ability of police law enforcement significantly impacts the effectiveness of on-site law enforcement, but there is currently no effective measurement tool available. This research aims to develop a police law enforcement psychological ability scale and test its reliability and validity.

**Methods:**

According to the principles and requirements of scale development, 239 law enforcement officers were selected as the study subjects. Then, following theoretical construction, scale development and testing, project analysis, exploratory factor analysis, confirmatory factor analysis, reliability and validity testing, and other procedures, a police law enforcement psychological ability scale was developed.

**Results:**

The new scale includes three dimensions-cognitive characteristics, emotional capacity, and volition quality-and nine sub-dimensions: attention, thinking, perception, emotion recognition, emotion control, emotion application, decisiveness, resilience, and bravery. The total correlation coefficient of the scale questions ranged from 0.670 to 0.791, and there is a significant positive correlation (*P* < 0.01). Exploratory factor analysis showed that the KMO test value was 0.954, and the Bartlett sphericity test result had a chi-squared value of 9717.356 (degree of freedom = 861, *P* < 0.01). The overall internal consistency coefficient of the scale is 0.978, and the coefficients of the cognitive characteristics, emotional capacity, and volition quality subscales are 0.946, 0.962, and 0.937, respectively. Confirmatory factor analysis showed that χ^2^/df < 3, RMSEA = 0.09, RFI = 0.765, IFI = 0.841, CFI = 0.840, NFI = 0.779, TLI = 0.830, and PNFI = 0.733.

**Conclusion:**

The model meets the requirements of psychometrics in terms of reliability and validity within an acceptable range and can be used for measuring and evaluating the psychological ability level of police law enforcement.

## 1 Introduction

Police work is considered a high-pressure and high-intensity job due to its unique working environment and targets (Blagden, 2014). Police law enforcement refers to the law enforcement activities of the police to stop and combat various illegal and criminal activities (Yu, 2014). It refers to the emergency policing of public security organs and their police officers to maintain national security and social order, protect the safety of people's lives and property, and respond to emergencies and rescue by the law (Zhao, 2005). In recent years, the social security situation has been complex and ever-changing, with various violent crimes and violent attacks on police officers increasing day by day. Police officers are facing more and more verbal and physical conflicts, and on-site psychological quality has become an important factor affecting their physical fitness, technical tactics, and language behavior (Li, 2019).

Psychological ability is a direct reflection of psychological qualities. Psychological ability primarily refers to psychological characteristics such as psychological control, ambition level, sense of responsibility, and the ability to cope with difficulties displayed in a certain psychological structure for a series of information processing, handling, and integration (Zhou and Li, 2000). Police psychological ability refers to a police officer's ability in cognition and psychological adaptation, which is an important component of psychological quality. In other words, it is the cognitive and psychological adaptation ability for the work environment and work of police officers and is the ability required in the three basic psychological activities of cognition, emotion, and volition (Bao, 2016). Undoubtedly, psychological ability is also an important psychological quality in police law enforcement effectiveness, playing a bridging role in psychological activities.

Law enforcement is also known as practical policing. Research on the psychological ability of law enforcement has been widely carried out in different disciplines and fields such as sports, fire protection, civil aviation, military, and police training and has achieved certain research results (Chen, 2009; Jin et al., 2018; Li et al., 2018; Zhao et al., 2018; Zhou et al., 2007). Studies by scholars have shown that psychological training is an important way to improve the efficiency of police law enforcement. Law enforcement psychology is defined as a special form of psychological existence, which is a special reflection of the police on the law enforcement environment in law enforcement activities. It is divided into three constituent factors: law enforcement cognition, emotion, and volition (Xu, 2007).

Currently, research on the structure and measurement tools of police psychological abilities is focused on the development of scales or questionnaires in various aspects, such as police psychological resilience (Zuo, 2013), police professional psychological quality (He et al., 2019), and professional competence psychological qualities of public security police officers (Wang, 2021). The above studies all originated from a certain concept and researched the development and application of measurement scales based on the characteristics of the police profession, which, to some extent, promoted the conduct of research on the evaluation and training of police professional psychological abilities. However, there is still a lack of mature and effective measurement tools for police law enforcement psychological abilities. Currently, the psychological ability of the police force, especially frontline law enforcement officers, in law enforcement is not yet clear. If this issue is not addressed, it will have a significant impact on the development of a psychological training system for law enforcement readiness, as well as the effectiveness and safety of law enforcement.

Therefore, this study sought to explore the structure of police law enforcement psychological abilities and to develop a standardized scale for police law enforcement psychological abilities, laying the foundation for the development of law enforcement psychological measurement tools and deepening research in the field of law enforcement psychology.

## 2 Materials and methods

### 2.1 Subjects

We randomly selected the police officers from different Public Security Bureaus in an eastern city of China, all of whom provided consent for enrollment. This study was approved by the Ethics Committee of the Shanghai Police College. A total of 261 online questionnaires were distributed and collected online through the “Questionnaire Star” platform, including 223 male policemen and 38 female policemen, all of them are front-line law enforcement policemen without religious beliefs. Informed consent was obtained from all the participants (at the start of the online questionnaire). Inclusion criteria: voluntary participation, complete completion of the questionnaire and passing the lie detector test. Exclusion criteria: incomplete questionnaire or failure to pass the lie detector test. According to the statistics of lie-detection questions, 239 valid questionnaires were obtained, with an effective rate of 92%. The test object situation is shown in Table 1.

**Table 1:** Basic information of the subjects (*n* = 239).

Bureau	Security patrol	Criminal investigation	Economic investigation	Traffic management	Prison management	Subway management	Community security	Other
Sample/*n*	113	14	7	14	30	10	48	3
Age/years	33.4 ± 4.61	34.2 ± 5.27	37.4 ± 5.13	35.4 ± 4.44	32.4 ± 4.11	33.6 ± 4.33	36.6 ± 2.87	33.3 ± 3.92
Years of police service/years	7 ± 2.95	8 ± 2.72	10 ± 4.81	9 ± 3.32	7 ± 3.17	8 ± 3.55	9 ± 4.65	8 ± 3.54

### 2.2 Study design and procedure

The scale's development was divided into two stages. The first stage involved the establishment of the scale structure, including item development, content validity, item analysis, and exploratory factor analysis to refine the scale. Then, during the second stage, reliability and validity tests were conducted on the revised scale. Data statistics were analyzed using the SPSS 22.0 and AMOS 23.0 statistical analysis software programs (IBM Corp., Armonk, NY, USA), encompassing correlation analysis, exploratory factor analysis, confirmatory factor analysis, and reliability and validity testing. All methods were carried out in accordance with relevant guidelines and regulations.

### 2.3 Review of the fundamentals of scale compilation

Psychology divides psychological processes into cognitive processes, emotional processes, and volitional processes (Lin et al., 2003). Due to the lack of clear research on measurement tools for law enforcement psychological ability, based on previous research on law enforcement psychological abilities, we sought to explore the specific dimensions of law enforcement psychological ability from the three constituent factors of cognition, emotion, and volition. Cognition is a necessary prerequisite for the generation of emotions (Lazarus, 1982), which has a direct impact on emotions and plays a regulatory role (Cui, 2019). Meanwhile, changes in emotions and the control of will complement the effects of behavior (Sun, 2020; Zhang et al., 2020). These three constitute a dialectical relationship between factual, value, and behavioral relationships (Hao and Hai, 2012). Therefore, exploring the psychological ability of police officers in law enforcement plays an important role in improving law enforcement quality and enhancing law enforcement effectiveness in practical behavior. Existing research on cognitive, emotional, and volitional dimensions is relatively extensive, providing a certain research foundation for this study.

#### 2.3.1 Research on cognitive dimensions

In the field of sports, Dong (2007) established a sports cognition level scale that includes four aspects—sports imagery, attention ability, sports perception, and thinking ability—in response to the development of sports cognition in modern sports psychology and training. Luo et al. (2011) combined cognitive psychology with motor intelligence, dividing motor intelligence into a part of cognitive psychological ability, and developed a motor intelligence assessment scale, which is divided into five dimensions: attention, memory, thinking, imagination, and perception (Luo et al., 2011). In the field of police discipline, this is similar to the viewpoint proposed by scholars in the field of police training regarding police law enforcement intelligence. Wang (2021) developed a questionnaire on the psychological qualities of law enforcement officers' professional competence from the perspective of professional competence. Here, the cognitive characteristics of police officers were divided into five aspects: attention, memory, thinking, imagination, and self-awareness (Wang, 2021). Studies have indicated that situational awareness and visual attention training can improve threat detection time and decision-making performance in law enforcement situations (Heusler and Sutter, 2022). The above research provides reference and inspiration for determining the cognitive ability feature dimensions in the psychological ability structure of police law enforcement.

#### 2.3.2 Research on the emotional dimension

In the late 1990s, psychologist Daniel Goleman defined emotional intelligence as “the ability to recognize one's own and others' feelings, self-motivation, good self-control, and emotional ability in interpersonal communication.” Emotional intelligence consists of five parts—namely, self-awareness, discernment and judgment of others' psychological states, self-emotional regulation and adjustment, self-motivation and self-efficacy, and social affinity. Wong and Law (2002) developed the emotional intelligence scale, which divides emotional intelligence into four subscales: the ability to evaluate and express one's own emotions, the ability to recognize and evaluate others' emotions, the ability to manage one's own emotions, and the ability to apply emotions. Qi (2005) pointed out that the emotional intelligence of police can affect the normal performance of police functions and summarized the elements of police emotional intelligence to be as follows: recognizing and identifying one's own emotions, effectively managing one's own emotions, judging and identifying the emotions of work objects, self-motivation, and emotional integration with others. Studies have shown that improving the emotional regulation skills of police officers can help them remain calm and focused (Bressler et al., 2023). In addition, the 24 questions in the neuroticism dimension of the Eysenck adult questionnaire, known as the emotional stability dimension, can reflect the degree of emotional stability of its respondents.

#### 2.3.2 Research on the volition dimension

According to previous research, it is generally believed that the basic qualities of volition include consciousness, decisiveness, persistence, and self-control, and each field has been further subdivided based on the above dimensions. In the field of sports, Liang et al. (2005) developed the advanced athlete volition scale, which divides volition into six dimensions: resilience, tenacity, decisiveness, self-control, goal clarity, and self-confidence. Li et al. (2011). proposed eight dimensions of the structure of athletes' volition: purposefulness, independence, decisiveness, bravery, resilience, self-control, self-awareness, and self-confidence. The volition quality model constructed by Li (2008) has two levels of dimensions, including four first-level dimensions and nine second-level sub-dimensions. Among these, the four first-level dimensions are self-awareness, independence, decisiveness, and resilience, while the nine second-level sub-dimensions include self-actualization desire, goal clarity, self-control, belief confirmation, tenacity, the concentration of intelligence, timely decision-making, fatigue endurance, and difficulty tolerance (Li, 2008). Meanwhile, in their work, Hao and Zhang (2013) compiled a police volition scale from four dimensions: self-awareness, decisiveness, resilience, and self-control.

**Figure 1:**
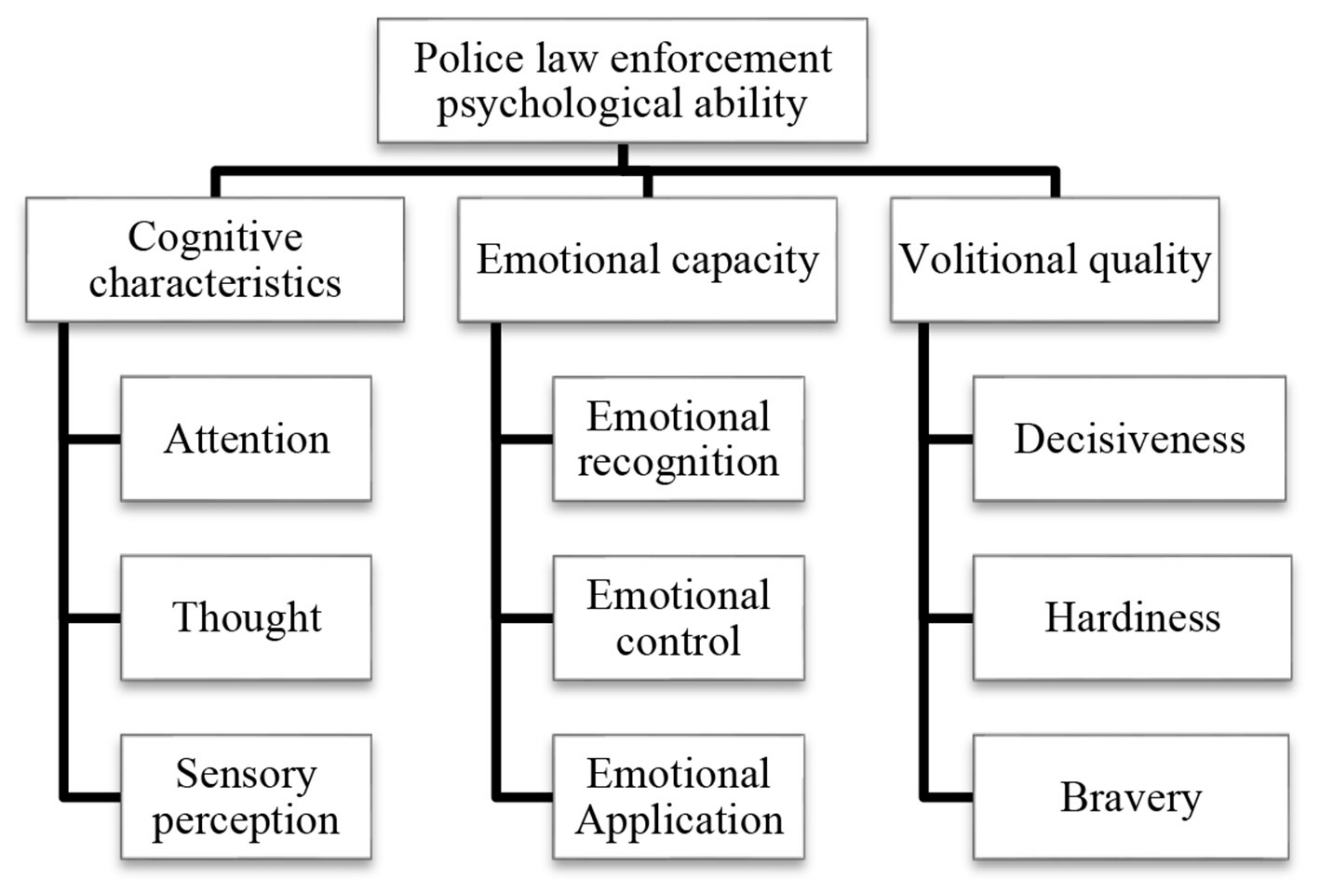
Framework diagram of police law enforcement psychological ability model.

By analyzing, researching, and drawing on previous experiences and results, based on the existing definition of police law enforcement psychology, this paper deeply analyzes the characteristics of law enforcement psychology as a psychological process and combines expert interview opinions and consulting suggestions to construct a structural model framework for police law enforcement psychological ability, as shown in Figure 1.

### 2.4 Scale compilation and formation

#### 2.4.1 Preparation and structure of the test items

According to the framework of the police law enforcement psychological ability model, the dimensions could be divided based on existing psychological research (Zhang, 2000), Based on the characteristics of urgency, danger, heaviness, and confrontation in the practical law enforcement tasks of public security police officers, various dimensions of police law enforcement psychological ability have been determined, and their meanings have been annotated (Table 2). An independent theoretical framework for the development of the scale has been designed, and entries for three subscales—namely, cognitive characteristics, emotional ability, and volitional quality—have been compiled. After collection, writing, and editing, a total of 56 questions were initially proposed. Two psychology experts and four police psychology teachers modified and screened the questions, then analyzed them based on the actual law enforcement work of frontline police officers. A total of nine questions that were repetitive, ambiguous, or did not reflect the measurement purpose well were deleted, leaving 47 items, including two lie detector questions, to form the police law enforcement psychological ability scale.

**Table 2:** Dimensions and meanings of the police law enforcement psychological ability scale.

First-level dimension	Secondary dimension	Connotation
Cognitive characteristics	Attention	The stability, anti-interference ability, or degree of concentration of police attention in law enforcement confrontation.
	Thought	An operational thinking activity ability that explores the relationship and patterns between police tactics, actions, and objects of operation.
	Sensory perception	The specialized perception of police law enforcement is a comprehensive ability formed by police officers in practical training and law enforcement practice, which enables them to keenly identify and perceive environmental clues.
Emotional capacity	Emotional recognition	Accurately identify and judge changes in the emotions of oneself and law enforcement targets
	Emotional control	When law enforcement encounters obstacles or provocations, police officers control their emotions.
	Emotional application	The ability of police officers to adjust their emotions toward constructive behavior and individual performance
Volitional quality	Decisiveness	Timely, firm, and non-blind adoption and execution of decisions
	Hardiness	Perseverance through hardship, exhaustion, difficulty, and pain, as well as unyielding perseverance through failure
	Bravery	Physical, social, moral, and creative courage

The compilation of this scale borrowed the overall format of existing measurement methods, modified and replaced it with specific scenario content items related to police law enforcement, borrowed the general form of an existing scale, and changed the content of the items (Wiersma and Jurs, 2004).

The scale is presented in first-person format. This closed-ended scale's development was based on the responses or statements of the participants in the interview communication, and it was recommended to use language that is similar, simple, and easy to understand to describe the law enforcement tasks and practical situations of public security police officers. Besides, using scales or research questionnaires recognized by the academic community, to make the content as comprehensive as possible, and to avoid missing any issues related to police law enforcement (Liu et al., 2016). The scale is scored according to the Likert 5-point scoring method, which awards 1, 2, 3, 4, and 5 points for complete non-conformity, basic non-conformity, unclear explanation, basic conformity, and complete conformity, respectively. We designed both positive and negative statement questions in the project description to prevent arbitrary responses. The initial project arrangement was not based on a scale, and the positive and negative statements were randomly shuffled, with two lie-detection questions inserted.

## 3 Results

### 3.1 Item analysis

The project analysis was mainly conducted in two stages. One step was to conduct an independent-samples *t*-test on the high and low groups. Using the highest 27% and lowest 27% of the total test scores as the dividing line between the high and low groups, statistical analysis revealed that, except for question 43 “when the chance of success is slim, I often give up law-enforcement tasks,” all other questions showed significant differences between the high and low groups (*P* < 0.05), indicating good discrimination ability. Therefore, question 43 needed to be deleted and the rest retained. The second step was to calculate the correlation coefficient between the question items and the total score of the scale. Statistical analysis showed that, except for questions VQ 32, VQ 37, and VQ 43, the correlation coefficients between the remaining items and the total score ranged between 0.670 and 0.791 and showed a significant positive correlation (*P* < 0.01), which should be retained. Therefore, we deleted questions VQ 32, VQ 37, and VQ 43.

### 3.2 Exploratory factor analysis

After analyzing the project, the remaining 42 questions were subjected to exploratory factor analysis. The KMO test value was 0.954 (*P* < 0.01), indicating that the sample data were suitable for factor analysis (Table 3).

**Table 3:** Suitability test of factor analysis.

KMO		0.954
Bartlett	Chi-square	9,717.356
	df	861
	sig	0.000

**Figure 2:**
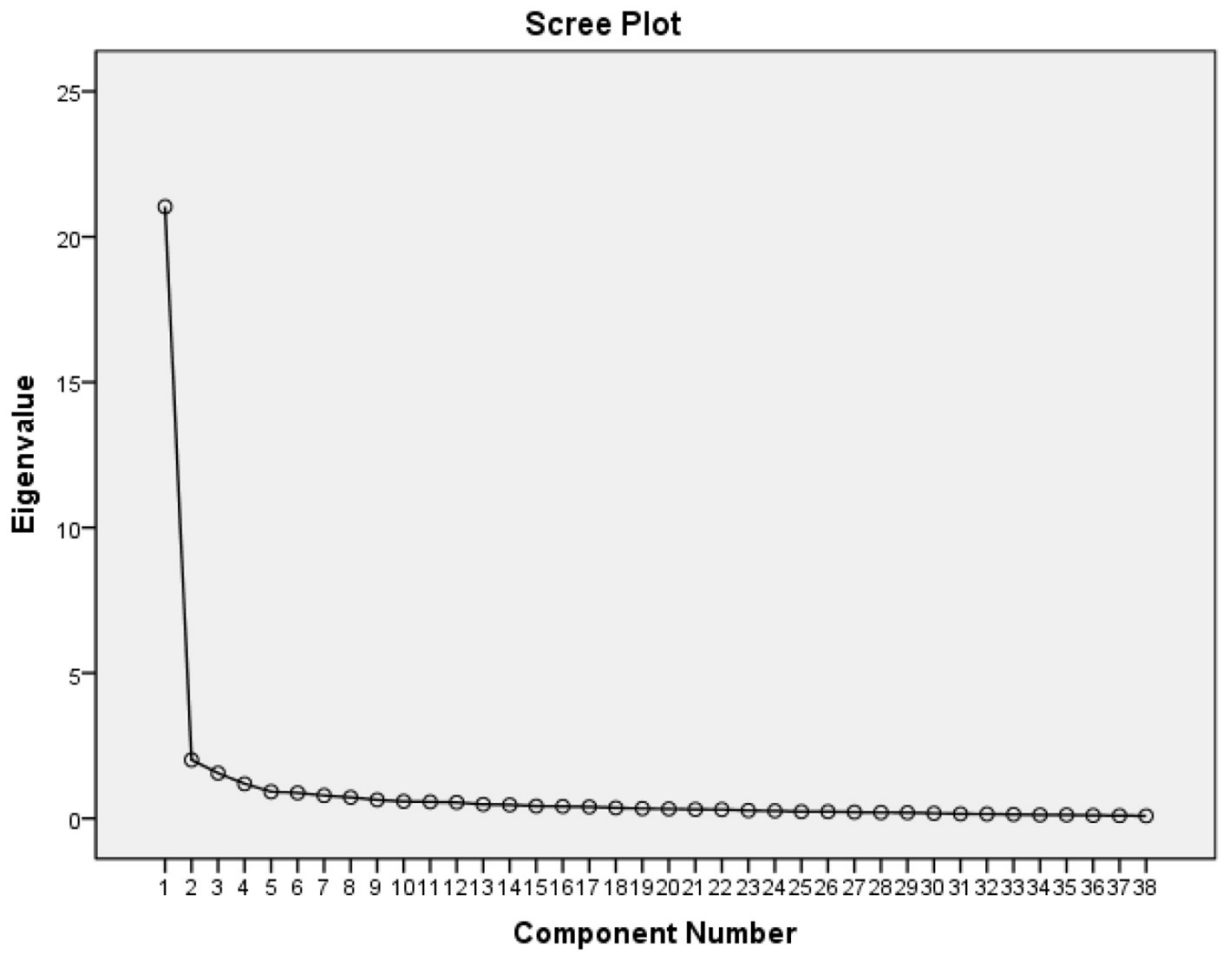
Scree plot of police law enforcement psychological ability.

According to the police law enforcement psychological ability model, three factors were limited, and four questions with load values greater than 0.5 appearing in more than one factor were deleted, including CC 10, CC 11, CC 13, and VQ 33. The remaining 38 questions all met the criteria of factor loading >0.5, and the content of the questions was consistent with the connotation of the factors, as shown in Table 4. The extracted eigenvalues of three factors were 23.417, 2.103, and 1.599, corresponding to emotional capacity, cognitive characteristics, and volitional quality, respectively. The cumulative explanatory power is 64.568% of the total variance, as shown in Table 5. The scree plot also reflects that the curve tends to stabilize from the third factor onwards, as shown in Figure 2.

**Table 4:** Summary table of factor load statistics for each dimension.

Items	Factor 1	Factor 2	Factor 3
EC 23	0.758		
EC 22	0.746		
EC 24	0.746		
EC 17	0.705		
EC 28	0.697		
EC 29	0.684		
EC 16	0.660		
EC 26	0.653		
EC 20	0.648		
EC 18	0.647		
EC 27	0.633		
EC 25	0.613		
EC 31	0.579		
EC 30	0.532		
EC 19	0.521		
CC 6		0.793	
CC 5		0.763	
CC 8		0.725	
CC 4		0.720	
CC 9		0.713	
CC 12		0.697	
CC 3		0.654	
CC 15		0.643	
CC 7		0.615	
CC 2		0.615	
CC 14		0.615	
CC 1		0.535	
VQ 44			0.764
VQ 45			0.728
VQ 46			0.681
VQ 47			0.663
VQ 36			0.658
VQ 42			0.650
VQ 34			0.646
VQ 41			0.606
VQ 40			0.604
VQ 35			0.575
VQ 39			0.555

**Table 5:** Factor eigenvalues and variance contribution rate.

Factor	Initial eigenvalue			Extract the sum of squared loads			Sum of squared rotational loads		
	Total	Variance percentage	Accumulate %	Total	Variance percentage	Accumulate %	Total	Variance percentage	Accumulate %
1	23.417	55.755	55.755	23.417	55.755	55.755	9.952	23.694	23.694
2	2.103	5.006	60.761	2.103	5.006	60.761	9.166	21.824	45.518
3	1.599	3.807	64.568	1.599	3.807	64.568	8.001	19.050	64.568

### 3.3 Reliability and validity analysis

According to the internal consistency test of the scale, after deleting the questions, the total Cronbach's alpha coefficient was 0.978, and the coefficients of the cognitive characteristics, emotional capacity, and volitional quality subscales were 0.946, 0.962, and 0.937, respectively. According to the general requirements of scale measurement, a reliability coefficient of at least 0.7 indicates that the measurement result is reliable. Fifty people were randomly selected and retested after a 2-week interval. The retest reliability of both the total scale and the subscales was above 0.7, indicating that the scale has high reliability.

In terms of validity testing, Table 6 shows that the combined reliability values all meet the testing criteria of greater than 0.7, and the average variance extraction values of each factor range from 0.575 to 0.632, with all meeting the criterion of being greater than 0.5, indicating that the overall convergent validity of the scale is good (Fornell and Larcker, 1981; Purnomo, 2017). The heterogeneity elemental ratio ranges from 0.811 to 0.863, all of which meet the criteria of less than 0.9, indicating good discriminant validity of the scale (Gold et al., 2001; Henseler et al., 2015).

**Table 6:** Test results of convergent validity and discriminant validity.

Factor	HTMT			CR	AVE
	CC	EC	VQ		
CC	—	—	—	0.947	0.597
EC	0.835	—	—	0.963	0.632
VQ	0.811	0.863	—	0.937	0.575

### 3.4 Confirmatory factor analysis

**Figure 3:**
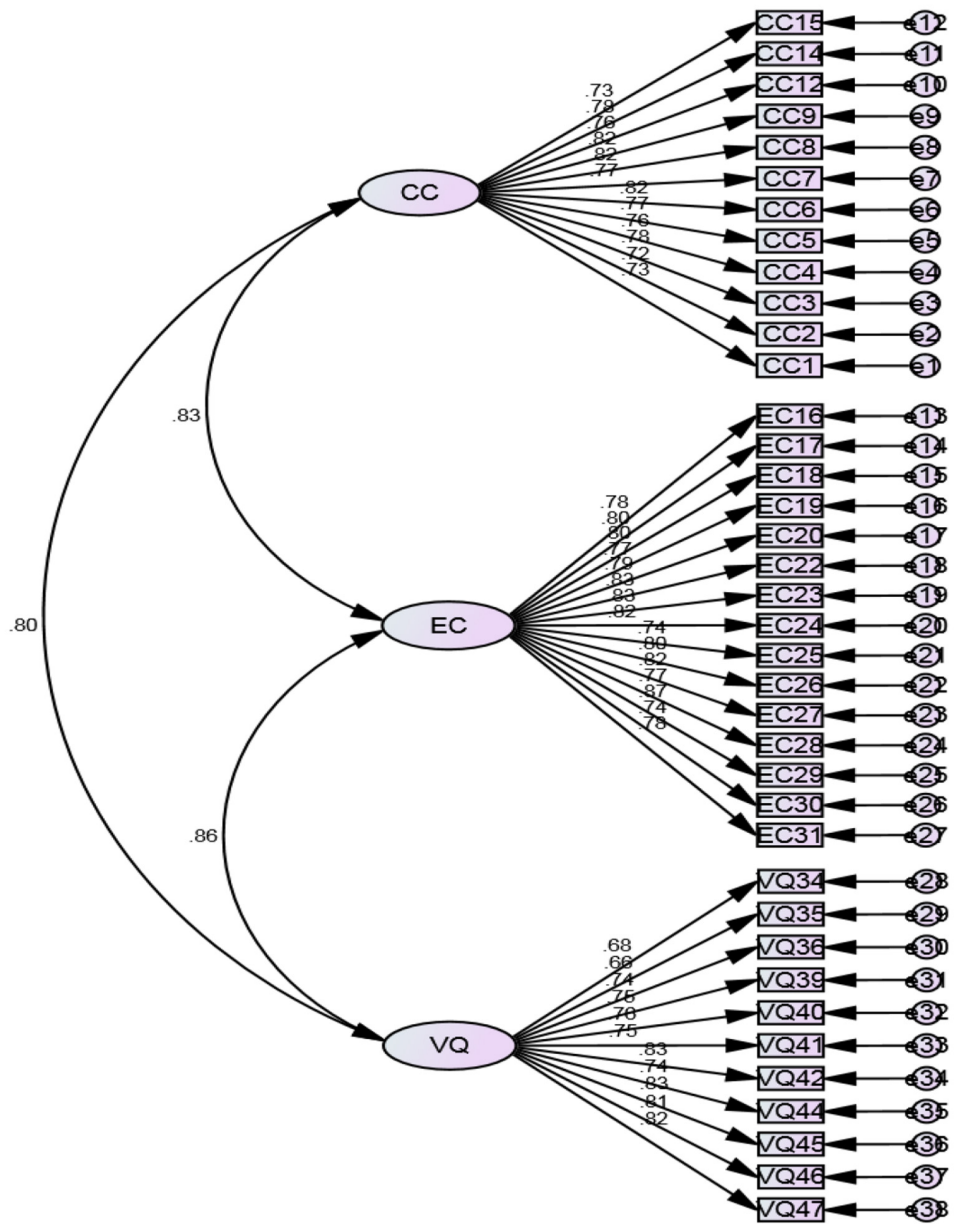
Confirmatory analysis structural model diagram.

Using AMOS 23.0 (IBM Corp., Armonk, NY, USA) for confirmatory factor analysis, with a chi-squared/df value of < 3 and RMSEA close to 0.08, the rest of the indicators can basically approach the level of 0.9. According to the overall results of the fitting indicators, as well as the sample size and model complexity, the model is within an acceptable range (Liu et al., 2016; Chang et al., 2022) (See Table 7 and Figure 3).

**Table 7:** Fitting indices of confirmatory factor analysis.

χ^2^	df	χ^2^/df	RMSEA	RFI	IFI	CFI	NFI	TLI	PNFI
1,974.787	662	2.983	0.091	0.765	0.841	0.840	0.779	0.830	0.733

## 4 Discussion

### 4.1 Model structure

This scale was developed using both top-down and bottom-up approaches (Qiao and Yu, 2014). First, we conducted a top-down theoretical construction. Based on reviewing and analyzing a large number of relevant literature and referring to widely recognized scales, both native and abroad, a conceptual model framework for the police law enforcement psychological ability scale was proposed, which reflects the psychological process characteristics of cognition, emotion, and volition. The cognitive characteristics of the police's law enforcement psychological ability reflect the ability of the public security police to observe the subtle actions of the suspect and his peers in law enforcement; perceive the possible hiding, attacking, or escaping behaviors of the suspect; and make reasonable and appropriate thinking, judgments, and analyses to showcase the corresponding decision-making ability. Good law enforcement cognitive ability is not only one of the standards for police mental health in the face of battle but also a key factor for police to complete law enforcement tasks and achieve law enforcement goals (Xu, 2007). Emotional ability refers to the ability of police officers to recognize the suspect's facial expressions and language; grasp the psychological state of the object that may resist or obey; and maintain rational and calm control over the object's verbal abuse, provocation, and physical confrontation. Studies have shown that the emotional stability of police is significantly higher than that of ordinary people (Skoglund et al., 2023). They can understand and use their positive emotions to employ appropriate coercive measures to achieve law enforcement goals, improve the efficiency of law enforcement in combat, and avoid excessive law enforcement (Cui, 2019). The quality of volition reflects the ability of police officers to fearlessly face danger, overcome difficulties, and decisively take corresponding coercive measures in emergency, difficult, dangerous, or heavy combat law enforcement situations (Xu, 2005). Second, the process is to collect practical law enforcement materials from the bottom up. The sources of the question items are twofold. Items were generated by referring to the expression of the question items in mature psychological scales. In addition, they were created by collecting police law enforcement scenarios through open-ended questionnaires, group discussions, and in-depth interviews; integrating the question items with the law enforcement scenarios; and revising the question items by psychological experts and police psychology teachers. Finally, 10 frontline police officers were asked to test the applicability of the questionnaire. Therefore, it ensures the good content validity of the scale.

### 4.2 Scale structure

Some items were deleted maybe because their wording was not closely related to the police combat scenario or there was a certain tendency to misunderstand the wording. In terms of reliability and validity, the internal consistency reliability of the overall scale is 0.978, and the internal consistency reliability of each dimension is above 0.9, indicating that the reliability of the scale is good. The correlation coefficient between the items and the total score is between 0.670 and 0.791 (*P* < 0.01), indicating good construct validity of the scale. The combination reliability value, mean variance extraction value, and heterogeneity element ratio of the scale are all good, indicating that the convergent validity and discriminant validity of the scale meet the testing requirements. Confirmatory factor analysis shows that all statistical measures and fitting indices are within an acceptable range (Fornell and Larcker, 1981; Wen et al., 2004). This indicates that the predictive validity of this scale is acceptable and can be used as an effective tool for evaluating the psychological ability of police law enforcement.

### 4.3 Scale scoring instructions

This scale is divided into three dimensions: law enforcement cognitive characteristics, law enforcement emotional ability, and law enforcement volition. In addition, there are nine sub-dimensions (attention, thinking, perception, emotion recognition, emotion control, and emotion application), with 38 items and two lie detector questions. Among them, in the cognitive dimension, “attention” includes questions 1, 9, and 10; “thinking” includes questions 3, 4, 6, 7, and 11; and “perception” includes questions 2, 5, 8, and 12. In the emotional dimension, “emotion recognition” includes questions 13, 15, 17, 20, and 21; “emotional control” includes questions 14, 19, 24, 25, and 27; and “emotional application” includes questions 16, 22, 23, 26, and 28. In the dimension of volition, “decisiveness” includes questions 30, 33, and 35; “resilience” includes questions 29, 38, and 39; and “bravery” includes questions 31, 34, 36, 37, and 40.

## 5 Conclusions

This research aims to develop a scale that can measure the psychological ability of police law enforcement. The study found that the police law enforcement psychological ability scale is divided into three dimensions, nine sub-dimensions, and 38 items. It uses the Likert 5-point scoring method, and its reliability and validity meet the requirements of psychometrics. It can be used for measuring and evaluating police law enforcement's psychological ability, which can help to fill gaps in the evaluation of police law enforcement's psychological ability.

## 6 Limitations

This research scale was developed based on police sample data of different ages and types of police units and can be widely used to assess the psychological abilities of law enforcement officers. However, as there is currently no scale related to police law enforcement psychology that can be used for criterion validity testing, this study did not verify the criterion validity. Subsequent field interviews and research can provide criterion standards for the scale developed in this study (Chen and Xue, 2022). In addition, self-assessment scales may have issues, such as social approval bias and reaction set, which may lead to overestimation of scores to a certain extent (Zheng, 2015). Therefore, future research can explore the development of other evaluation scales based on this or conduct in-depth screening of the law enforcement psychological abilities of outstanding and average performers in order to more accurately evaluate the advantages and disadvantages of police law enforcement psychological abilities.

## 7 Application

The options and scoring criteria are as follows: one point for “complete non-conformity,” two points for “basically non-conformity,” three points for “unclear explanation,” four points for “basically conformity,” and five points for “completely conformity.” The average or total score can be calculated for each dimension. Questions 18 and 32 are lie-detection questions and are not included in the scores of each dimension. If the total score of the two questions for the subject is ≥9 points, the subject's questionnaire must be excluded. Please refer to the attachment for the items on the scale.

## Data Availability

The raw data supporting the conclusions of this article will be made available by the authors, without undue reservation.
