# Supplementary material for: Development of police law enforcement psychological ability scale and test of reliability and validity
Source: Front Psychol. 2026 May 7;17:1811849. doi: 10.3389/fpsyg.2026.1811849 (PMC13189811; doi:10.3389/fpsyg.2026.1811849)
Supplement: Supplementary file 1 [file Table_1.docx]

**Police Law Enforcement Psychological Ability Scale**

Please choose the police law enforcement situation described in the following questions that best suits your own situation from the corresponding positions of "completely disagree -1", "basically disagree -2", "unclear -3", "basically agree -4", and "completely agree -5".

1. I usually notice every move of the suspect.

2. I can sense the speed of the suspect's attack.

3. I often analyze the gains and losses of police skills and actions in law enforcement cases.

4. I often engage in reverse thinking in law enforcement scenarios.

5. I can sense where the suspect may be hiding.

6. I can quickly make corresponding adjustments based on on-site changes.

7. I have a clear understanding of my technical and tactical abilities.

8. I can perceive the rationality of my own technical and tactical use.

9. I am good at observing the details of some law enforcement practical scenes.

10. The external pressure will not affect my law enforcement actions.

11. I am good at analyzing my tactical ideas.

12. I can sense the time spent confronting the suspect.

13. I usually know the reasons why I have certain feelings.

14. When law enforcement encounters provocation, I can control my temper.

15. I understand the emotions of the onlookers very well.

16. I am usually able to set law enforcement task goals for myself and strive to achieve these goals as much as possible.

17. I am well aware of my emotions.

18. I can perceive all the psychology and emotions of the suspect.

19. I have a strong ability to control my emotions.

20. I can usually guess the emotions of suspects from their behavior.

21. I can keenly perceive the feelings and emotions of suspect.

22. I often tell myself that I am a person with law enforcement abilities.

23. I often encourage myself to do my best.

24. I am very good at controlling my emotions during law enforcement confrontations.

25. When I am angry, I can usually calm down in a short amount of time.

26. In the face of setbacks, I keep myself in a good mood to cope with challenges.

27. My emotions are generally not affected by the environment when under pressure.

28. I am someone who can encourage myself.

29. I can endure extreme fatigue to complete law enforcement tasks.

30. At critical moments in law enforcement battles, apart from winning, little else is thought of.

31. I dare to fight against the attacks of criminals.

32. In the face of any unexpected situation, I am able to successfully complete law enforcement tasks.

33. In emergency situations, my mind is exceptionally clear.

34. Those who know me think I am a brave person.

35. If necessary at critical moments in law enforcement actions, I will immediately use corresponding coercive measures (verbal, bare-handed, equipment or weapons).

36. I am able to face danger without fear.

37. I will spare no effort to complete law enforcement tasks.

38. I am able to persistently achieve all the goals that have been set.

39. In the toughest and most tiring times, I often grit my teeth and endure.

40. Even if faced with strong protests and accusations from onlookers, as long as legal procedures are followed, I will bravely take law enforcement actions.
